# Short- and long-term outcomes of robotic-assisted versus video-assisted thoracoscopic lobectomy in non-small cell lung cancer patients aged 35 years or younger: a real-world study with propensity score-matched analysis

**DOI:** 10.1007/s00432-023-04933-6

**Published:** 2023-05-30

**Authors:** Hanbo Pan, Jiaqi Zhang, Yu Tian, Ningyuan Zou, Hongda Zhu, Zenan Gu, Weiqiu Jin, Junwei Ning, Long Jiang, Jia Huang, Qingquan Luo

**Affiliations:** 1 Shanghai Lung Cancer Center, Department of Thoracic Surgical Oncology, Shanghai Chest Hospital, Shanghai Jiao Tong University School of Medicine, Shanghai, China; 2 Department of Thoracic Surgery, Tongren Hospital, Shanghai Jiao Tong University School of Medicine, Shanghai, China

**Keywords:** Non-small cell lung cancer, Robotic-assisted thoracoscopic surgery, Video-assisted thoracoscopic surgery, Perioperative outcomes, Lymph node assessment, Long-term survival

## Abstract

**Purpose:**

This study compared short- and long-term outcomes of robotic-assisted thoracoscopic surgery (RATS) versus video-assisted thoracoscopic surgery (VATS) for lobectomy in young adults aged ≤ 35 years with non-small cell lung cancer (NSCLC), aiming to assess the superiority of RATS over VATS for this special group of patients.

**Methods:**

A total of 1355 consecutive NSCLC cases aged 18–35 years undergoing RATS (*n = *105) or VATS (*n = *1250) between 2014 and 2021 were retrospectively identified from a prospectively maintained database. Propensity score matching (PSM) was applied to establish a 1:3 RATS versus VATS ratio. Baseline clinicopathological characteristics, perioperative outcomes, lymph node (LN) assessment, and long-term survival were investigated.

**Results:**

Following PSM, 105 and 315 cases were in the RATS and VATS groups, respectively. RATS led to a shorter postoperative hospital stay than VATS (4.0 ± 1.5 *vs* 4.3 ± 1.7 days, *p = *0.02). The two groups were comparable in other perioperative outcomes and postoperative complications (all *p > *0.05). Moreover, RATS assessed more LNs (9.4 ± 4.4 *vs* 8.3 ± 3.6, *p = *0.03), especially N1 LNs (4.2 ± 3.1 *vs* 3.5 ± 2.2, *p = *0.02), than VATS. By comparison, no difference in 5-year recurrence-free survival (RFS), overall survival (OS), or recurrence or mortality patterns was found between the two groups (all *p > *0.05). Further subgroup analyses also observed similar long-term outcomes between the two groups regarding age, gender, and smoking history. Finally, Cox’s analyses found that the surgical approach was not independently correlated with RFS or OS.

**Conclusion:**

RATS shortened postoperative hospital stay, assessed more N1 and total LNs, and achieved comparable long-term outcomes to VATS for very young NSCLC patients.

**Supplementary Information:**

The online version contains supplementary material available at 10.1007/s00432-023-04933-6.

## Introduction

Non-small cell lung cancer (NSCLC) is one of the most common malignancies and has become a major public health problem in the world (Siegel et al. 2023). NSCLC typically affects elderly individuals with a median age at the first diagnosis of approximately 70 years, and younger adults are uncommon to suffer from primary NSCLC (Garrana et al. 2021). Nevertheless, with the increased implementation of thin-section thoracic computed tomography (CT) and advances in diagnostic modalities which effectively contribute to assessing early-stage disease, a growing number of young adults have been diagnosed with NSCLC in recent years, with the most prevalent histology being adenocarcinoma and the preponderance of female (Thai et al. 2021; Galvez-Nino et al. 2020; Sacher et al. 2016). Therefore, the optimal surgical approach is of critical importance for young patients, especially very young adults aged ≤ 35 years who may be associated with high reproduction and financial concerns, to achieve better oncological outcomes, emotional and psychological conditions, and quality of life (Galvez-Nino et al. 2020; Subramanian et al. 2010; Landwehr et al. 2016).

Although traditional thoracotomy is still the standard approach in treating NSCLC, minimally invasive surgery (MIS) technics have been widely applied in recent years. Video-assisted thoracoscopic surgery (VATS) is the most prevalent adopted MIS approach which reduces operation-related complications, accelerates postoperative recoveries, and leads to better life qualities compared with thoracotomy for early-stage NSCLC patients (Bendixen et al. 2016). Since its first performance in 2002, robotic-assisted thoracoscopic surgery (RATS) has attracted the growing interest of thoracic surgeons and is becoming increasingly prevalent in treating NSCLC as an alternative to thoracotomy and VATS (Huang et al. 2021). The robotic-assisted surgical system provides a high-definition, three-dimensional (3D), and 10–15 times magnified operation field and highly flexible robotic arms with an increased degree of motion and rotational freedom, allowing operators to perform the surgery more conveniently and accurately (Jin et al. 2022). Previous studies have compared the feasibility and oncological efficacy of RATS versus VATS for NSCLC, suggesting that RATS assessed more lymph nodes (LNs), reduced operation-related pain, led to a higher postoperative quality of life, and achieved similar long-term outcomes (Veronesi et al. 2016; Kneuertz et al. 2019). Therefore, RATS might be especially suitable for very young patients pursuing a quick postoperative recovery and high quality of life. However, RATS usually increased expenditures with its superiority over VATS concerning short- and long-term outcomes remaining unrevealed specified for young adults with NSCLC (Jin et al. 2022). Consequently, debate persists on applying this novel modality to this special group of patients.

In the present study, we retrospectively identified a large cohort of NSCLC patients aged ≤ 35 years undergoing RATS or VATS lobectomy from a prospectively maintained database and compared the perioperative outcomes, LN dissection, and long-term survival between the two surgical approaches, aiming to assess the advantages of RATS over VATS.

## Materials and methods

### Patient selection and data collection

**Fig. 1:**
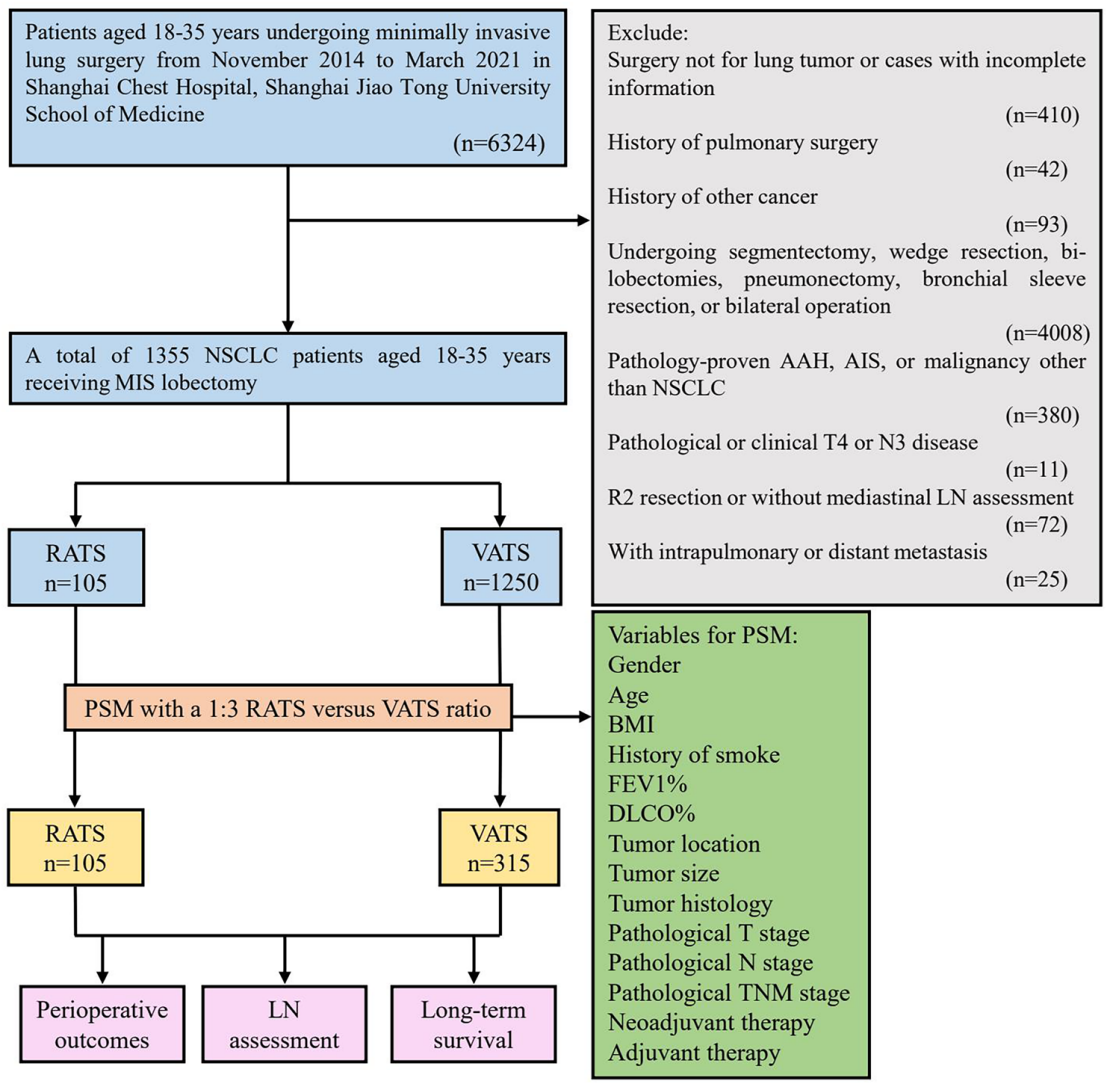
Flow chart of the study population. *NSCLC* non-small cell lung cancer, *AAH* atypical adenomatous hyperplasia, *AIS* adenocarcinoma in situ, *LN* lymph node, *MIS* minimally invasive surgery, *RATS* robotic-assisted thoracoscopic surgery, *VATS* video-assisted thoracoscopic surgery, *PSM* propensity score matching, *BMI* body mass index, *FEV1* forced expiratory volume in 1 s; *DLCO* diffusing capacity for carbon monoxide

We retrospectively identified NSCLC patients aged 35 years or younger who underwent RATS or VATS lobectomy in Shanghai Chest Hospital, Shanghai Jiao Tong University School of Medicine during the period of November 2014 and March 2021 (Fig. 1). The inclusion criteria were as follows: (1) receiving RATS or VATS lobectomy; (2) aged 18–35 years; (3) pathology-confirmed NSCLC. The exclusion criteria were as follows: (1) surgery not for lung malignancy or cases with incomplete information; (2) History of lung surgery; (3) History of malignancy; (4) Undergoing segmentectomy, wedge resection, pneumonectomy, bi-lobectomies, bronchial sleeve resection, or bilateral operation; (5) pathology-proven adenocarcinoma in situ (AIS) or malignancy other than NSCLC; (6) clinical/pathological T4 or N3 disease; (7) R2 resection (residual macroscopic tumor) or without mediastinal LN assessment; (8) preoperative intra-pulmonary or distant metastasis. A total of 1355 patients were finally enrolled and further divided into the RATS (*n = *105) and VATS (*n = *1250) groups. The following data were collected: (1) baseline clinicopathological characteristics such as gender, age, history of smoke, body mass index (BMI), preoperative comorbidities, pulmonary functions [% of forced expiratory volume in 1 s (FEV1%), and % of diffusing capacity for carbon monoxide (DLCO%)], arterial blood gas analysis [oxygen pressure (PaO2), oxygen saturation (SaO2), and carbon dioxide pressure (PaCO2)], surgical location, tumor size, pathological T, N, and TNM stage of the disease, induction therapy, and adjuvant therapy; (2) perioperative outcomes including surgical duration, conversion, reoperation, intraoperative bleeding, postoperative blood transfusion, postoperative ICU admission, duration and volume of chest tube drainage, length of postoperative hospital stays, and postoperative complications; (3) LN assessment including the N1, N2, and the total number of dissected LNs and LN stations; (4) long-term outcomes including 1-, 3-, and 5-year recurrence-free survival (RFS), overall survival (OS), and recurrence and mortality patterns. All cases were staged following the 8th edition of the TNM staging system of the International Association for the Study of Lung Cancer.

### Perioperative evaluation

Pulmonary and cardiac function tests were routinely performed before the operation to ensure the surgical tolerance of patients. For patients with impaired respiratory function symptoms or decreased FEV1, FEV1%, or DLCO% detected by the pulmonary function test, the arterial blood gas analysis was further performed. High-resolution thoracic CT and/or positron emission tomography/CT (PET/CT) were conducted to assess mediastinal and pulmonary LN status. PET/CT, bone scintigraphy, abdominal ultrasound, and enhanced cranial magnetic resonance imaging were carried out to determine distant metastasis.

### Surgical techniques

RATS and VATS were performed according to the procedure reported by our surgical team previously (Huang et al. 2021, 2018; Hou et al. 2022). All patients received general intravenous (*i.v.*) anesthesia with double-lumen intubation and single-lung ventilation managed by dedicated thoracic anesthesiologists. RATS was conducted by adopting the Da Vinci Surgical System (Intuitive Surgical, CA, USA) via four minimal incisions without spreading the ribs. A 30-degree three-dimensional (3D) endoscope was inserted through the camera port located on the posterior axillary line's 7th or 8th intercostal space. Two incisions were symmetrically made at the 7th and 9th intercostal spaces along the mid-axillary and infrascapular lines, respectively. A utility port was created at the 3rd or 4th intercostal space on the anterior axillary line for lung retraction, operating field exposure, and specimen retrieval. Conventionally, VATS was performed via three or four minimal incisions with the non-rib spreading technic. The camera port was created at the 6th or 7th intercostal space along the anterior axillary line. Two incisions were made at the 3rd or 4th, and 8th intercostal spaces on the anterior and posterior axillary lines, respectively. The fourth port was created at the 9th intercostal space along the posterior axillary line, if needed, for assistance. A radical lobectomy with systematic mediastinal LN dissection was carried out. The interleaf fissures were divided, and hilar structures were dissected and identified individually, followed by the dissection of the target pulmonary artery and vein, and bronchus using endoscopic staplers. The resection margin was evaluated by the intraoperative frozen section for all patients. After ensuring the absence of active bleeding in the thoracic cavity and air leakage from the bronchial stump, the incisions were closed with one or two 24F chest tubes left. The conversion was defined as the operation starting with RATS or VATS dissection and finishing as the rib spreading thoracotomy. The representative operation video of RATS lobectomy is provided in Supplementary Video 1.

### Postoperative management

All patients received the enhanced recovery protocol, including smoking cessation two weeks before surgery, breathing exercises, and early postoperative exercises. The 30 day postoperative complications were recorded, followed by being classified according to the Clavien–Dindo classification system as follows: I, any deviation from the ordinary postoperative course without requiring pharmacological treatment or surgical intervention or requiring drugs such as antipyretics, analgesics, diuretics, antiemetics, or electrolytes; II, complication requiring pharmacological intervention, including blood transfusion and total parenteral nutrition; III, comorbidities requiring operative or endoscopic intervention; IV, complication requiring ICU treatment; and V, death of the patient (Dindo et al. 2004). Specifically, pulmonary complications included pneumonia, acute respiratory distress syndrome (ARDS), respiratory failure requiring reintubation, empyema, and pulmonary embolism. Cardiac comorbidities included arrhythmia and myocardial ischemia or infarction. Anastomotic complications included prolonged air leaks, anastomotic dehiscence, and bronchopleural fistula. Other comorbidities included chylothorax, vocal cord paralysis, wound infection, and deep venous thrombosis.

The chest tube was removed when: (1) absence of apparent air leak and subcutaneous emphysema, 92) the drainage volume was less than 200 mL/day, 93) no cloudy, densely bloody, or purulent pleural effusion, and 4) the chest X-ray images indicated excellent resorption of the lung. Conventionally, patients were discharged the day or one day after removing drainage tubes unless there were comorbidities that still required intervention. Standard adjuvant therapy, including chemotherapy, immunotherapy, and radiotherapy, was recommended for patients if deemed necessary.

### Follow-up

The lifelong follow-up assessment was planned for all patients one month after the operation and varied afterward: patients with histology of MIA were evaluated annually, while the other patients were measured every three months for the first two years, every half year in years three–five, and annually afterward. For postoperative follow-up of patients, thoracic CT, routine blood tests, serum tumor markers tests, and ultrasound were conventional approaches. Additionally, PET–CT was applied if suspected recurrence or metastasis were indicated during follow-up assessment. Moreover, head MRI and bone scintigraphy were applied for patients with suspected metastasis specified to the brain or bone. Telephone or Internet follow-ups were conducted yearly for patients who did not regularly come to the outpatient clinic until death or March 2023. The latest electronic medical data were recorded for patients lost to follow-up. The radiological recurrence tumors were defined as multiple new pulmonary lesions with the consolidation-to-tumor ratio (the ratio of the size of the solid component to the overall tumor diameter) of > 0.5 for all nodules, following the guidelines of radiologists (Yotsukura et al. 2021). RFS was defined as the duration from operation to any local or distant tumor recurrence, and patients with non-cancer-related death were deemed event free. OS was defined as the duration from operation to death.

### Statistical analysis

Categorical variables were expressed using frequencies and percentages, while continuous variables were expressed using mean ± standard deviation (SD). To compare categorical variables, Pearson’s chi-square tests or Fisher’s exact tests were adopted. The homogeneity of variance and normality of distribution for continuous variables were determined using the Kolmogorov–Smirnov test. The student’s t-test was conducted for continuous variables with normal distribution and homogeneous variance, and the Mann–Whitney *U* test was performed otherwise. Kaplan–Meier curves log-rank (Mantel–Cox) test was carried out to analyze survival profiles. Cox hazard regression model analysis was further conducted to identify factors relevant to RFS and OS. To mitigate potential patient selection bias, propensity score matching (PSM) was carried out based on 14 critical baseline characteristics, including gender, age, history of smoke, BMI, FEV1%, DLCO%, surgical location, tumor size, tumor histology, pathological T stage, pathological N stage, pathological TNM stage, neoadjuvant therapy, and adjuvant therapy to establish a 1:3 RATS versus VATS ratio. SPSS version 26.0 (IBM Corporation, Armonk, NY, USA) was applied for statistical analysis and PSM, while GraphPad Prism-9 (GraphPad Software Inc., San Diego, CA, USA) was used for analyzing survival profiles. A two-tailed p value less than 0.10 was considered statistically significant for the Cox hazards regression model analysis, while less than 0.05 was considered statistically significant in all other tests.

## Results

### Baseline clinicopathological characteristics

The baseline clinicopathological characteristics of the included patients before PSM are shown in Table 1. Patients who received RATS or VATS were associated with a similar distribution of gender (*p = *0.90), age (32.2 ± 3.0 *vs* 32.0 ± 2.8 years, *p = *0.34), history of smoke (*p = *0.76), BMI (22.0 ± 2.8 *vs* 21.8 ± 4.4 kg/m^2^, *p = *0.62), preoperative comorbidities (*p = *0.59), FEV1% (96.8 ± 11.5 *vs* 96.3 ± 12.2, *p = *0.47), DLCO% (97.5 ± 13.5 *vs* 97.8 ± 16.9, *p = *0.72), surgical location (*p = *0.34), tumor histology (*p = *0.68), tumor size (16.7 ± 9.9 *vs* 17.4 ± 10.1 mm, *p = *0.69), pathological T (*p = *0.78), N (*p = *1.00), and TNM (*p = *0.99) stage of the disease, neoadjuvant therapy (*p = *1.00), and adjuvant therapy (*p = *0.75). For patients receiving blood gas analysis, the PaO_2_, SaO_2_, and PaCO_2_ indexes were all similar between the two groups (all *p > *0.05). PSM was then applied to establish a 1:3 RATS versus VATS ratio, and all baseline cofounding features of included cases were well balanced between the two groups following PSM (all *p > *0.05).

**Table 1:** Baseline clinicopathological characteristics of patients before and after PSM

Variables	Before PSM			After PSM		
	RATS (*n = *105)	VATS (*n = *1250)	*P* value	RATS (*n = *105)	VATS (*n = *315)	*P* value
Gender, *n* (%)						
Male	38 (36.2)	445 (35.6)	0.90	38 (36.2)	116 (36.8)	0.91
Female	67 (63.8)	805 (64.4)		67 (63.8)	199 (63.2)	
Age (years), mean ± SD	32.2 ± 3.0	32.0 ± 2.8	0.34	32.2 ± 3.0	32.2 ± 2.9	0.99
History of smoke, *n* (%)						
Ever	8 (7.6)	106 (8.5)	0.76	8 (7.6)	22 (7.0)	0.83
Never	97 (92.4)	1144 (91.5)		97 (92.4)	293 (93.0)	
BMI (kg/m^2^), mean ± SD	22.0 ± 2.8	21.8 ± 4.4	0.62	22.0 ± 2.8	22.0 ± 3.1	0.86
Comorbidities, *n* (%)						
Yes	6 (5.7)	89 (7.1)	0.59	6 (5.7)	21 (6.7)	0.73
No	99 (94.3)	1161 (92.9)		99 (94.3)	294 (93.3)	
Pulmonary function, mean ± SD						
FEV1% (of predicted)	96.8 ± 11.5	96.3 ± 12.2	0.47	96.8 ± 11.5	96.7 ± 10.5	0.61
DLCO% (of predicted)	97.5 ± 13.5	97.8 ± 16.9	0.72	97.5 ± 13.5	97.6 ± 13.9	1.00
Blood gas analysis^a^, mean ± SD						
PaO_2_ (mmHg)	92.6 ± 7.0	92.0 ± 8.4	0.55	92.6 ± 7.0	93.7 ± 8.0	0.63
SaO_2_ (%)	98.1 ± 1.7	97.4 ± 3.6	0.97	98.1 ± 1.7	98.3 ± 1.9	0.83
PaCO_2_ (mmHg)	41.3 ± 4.2	41.1 ± 4.6	0.94	41.3 ± 4.2	40.1 ± 4.0	0.48
Surgical location, *n* (%)						0.99
Left upper lobe	10 (9.5)	207 (16.6)	0.34	10 (9.5)	34 (10.8)	
Left lower lobe	26 (24.8)	245 (19.6)		26 (24.8)	81 (25.7)	
Right upper lobe	29 (27.6)	335 (26.8)		29 (27.6)	82 (26.0)	
Right middle lobe	17 (16.2)	182 (14.6)		17 (16.2)	50 (15.9)	
Right lower lobe	23 (21.9)	281 (22.5)		23 (21.9)	68 (21.6)	0.99
Tumor histology, *n* (%)			0.68			1.00
MIA	29 (27.6)	300 (24.0)		29 (27.6)	87 (27.6)	
Adenocarcinoma	71 (67.6)	879 (70.3)		71 (67.6)	215 (68.3)	
Others^b^	5 (4.8)	71 (5.7)		5 (4.8)	13 (4.1)	
Tumor size (mm), mean ± SD	16.7 ± 9.9	17.4 ± 10.1	0.69	16.7 ± 9.9	16.4 ± 10.8	0.48
Pathological T stage, *n* (%)			0.78			1.00
T1a(mi)	29 (27.6)	300 (24.0)		29 (27.6)	87 (27.6)	
T1a	19 (18.1)	215 (17.2)		19 (18.1)	58 (18.4)	
T1b	37 (35.2)	429 (34.3)		37 (35.2)	112 (35.6)	
T1c	8 (7.6)	97 (7.8)		8 (7.6)	22 (7.0)	
T2a	8 (7.6)	104 (8.3)		8 (7.6)	24 (7.6)	
T2b	3 (2.9)	69 (5.5)		3 (2.9)	9 (2.9)	
T3	1 (1.0)	36 (2.9)		1 (1.0)	3 (1.0)	
Pathological N stage, *n* (%)			1.00			1.00
N0	98 (93.3)	1154 (92.3)		98 (93.3)	294 (93.3)	
N1	3 (2.9)	40 (3.2)		3 (2.9)	9 (2.9)	
N2	4 (3.8)	56 (4.5)		4 (3.8)	12 (3.8)	
Pathological TNM stage, *n* (%)			0.99			1.00
IA1	48 (45.7)	515 (41.2)		48 (45.7)	145 (46.0)	
IA2	35 (33.3)	407 (32.6)		35 (33.3)	105 (33.3)	
IA3	6 (5.7)	79 (6.3)		6 (5.7)	18 (5.7)	
IB	5 (4.8)	82 (6.6)		5 (4.8)	19 (6.0)	
IIA	3 (2.9)	48 (3.8)		3 (2.9)	6 (1.9)	
IIB	4 (3.8)	56 (4.5)		4 (3.8)	9 (2.9)	
IIIA	4 (3.8)	57 (4.6)		4 (3.8)	12 (3.8)	
IIIB	0 (0.0)	6 (0.5)		0 (0.0)	1 (0.3)	
Neoadjuvant therapy, *n* (%)	4 (3.8)	53 (4.2)	1.00	4 (3.8)	13 (4.1)	1.00
Adjuvant therapy, *n* (%)	9 (8.6)	119 (9.5)	0.75	9 (8.6)	29 (9.2)	0.84

Continuous data are expressed as mean ± SD, and categorical data are shown as number (percentage). Bold indicates the statistically significant *p* value (*p* < 0.05). Blood gas analysis^a^: eight and seventy-nine patients in the RATS and VATS groups, respectively, received peripheral arterial blood gas analysis before PSM. Others^b^ squamous cell carcinoma, carcinoid tumor, mixed tumor, and mucosa-associated lymphoid tissue lymphoma. *RATS* robotic-assisted thoracoscopic surgery, *VATS* video-assisted thoracoscopic surgery, *SD* standard deviation, *BMI* body mass index, *FEV1* forced expiratory volume in 1 s, *DLCO* diffusing capacity for carbon monoxide, *PaO2* oxygen pressure, *SaO2* oxygen saturation, *PaCO2* carbon dioxide pressure, *MIA* minimally invasive adenocarcinoma.

### Perioperative outcomes and LN dissection

**Fig. 2:**
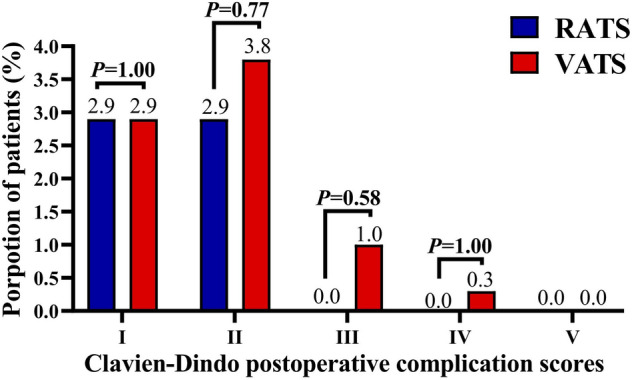
Comparison of 30-day Clavien–Dindo postoperative complication scores of NSCLC patients aged ≤ 35 years who underwent RATS or VATS. *RATS* robotic-assisted thoracoscopic surgery, *VATS* video-assisted thoracoscopic surgery

The perioperative outcomes of matched patients are expressed in Table 2. By comparison, RATS and VATS were associated with a similar surgical duration (91.4 ± 23.4 *vs* 94.3 ± 34.6 min, *p = *0.95), the incidence of conversion (*p = *1.00) and reoperation (*p = *1.00), intraoperative bleeding (82.38 ± 17.99 *vs* 84.24 ± 16.71 mL, *p = *0.35), and postoperative blood transfusion (*p = *1.00). Moreover, the RATS group had a shorter postoperative hospital stay (4.0 ± 1.5 *vs* 4.3 ± 1.7 days, *p = *0.02) than the VATS group. Additionally, no difference was found between the two groups in postoperative ICU admission (6.7% *vs* 9.2%, *p = *0.42) and chest tube drainage volume (686.9 ± 364.9 *vs* 701.8 ± 335.9 mL, *p = *0.41) and duration (3.4 ± 1.3 *vs* 3.5 ± 1.6 days, *p = *0.51), and postoperative complications (all *p > *0.05). Meanwhile, no operation-related mortality was found in the RATS or VATS groups. Finally, patients receiving RATS or VATS were associated with comparable Clavien–Dindo postoperative complication scores (Fig. 2).

**Table 2:** Perioperative outcomes and LN dissection of matched patient

Variables	RATS (*n = *105)	VATS (*n = *315)	*P* value
Surgical duration (mins), mean ± SD	91.4 ± 23.4	94.3 ± 34.6	0.95
Conversion to thoracotomy, *n* (%)	1 (1.0)	5 (1.6)	1.00
Reoperation, *n* (%)	0 (0.0)	2 (0.6)	1.00
Intraoperative bleeding (mL), mean ± SD	82.38 ± 17.99	84.24 ± 16.71	0.35
Postoperative blood transfusion, *n* (%)	1 (1.0)	3 (1.0)	1.00
ICU admission, *n* (%)	7 (6.7)	29 (9.2)	0.42
Chest tube drainage, mean ± SD			
Length (days)	3.4 ± 1.3	3.5 ± 1.6	0.51
Volume (mL)	686.9 ± 364.9	701.8 ± 335.9	0.41
Postoperative hospital stays (days), mean ± SD	4.0 ± 1.5	4.3 ± 1.7	**0.02**
Postoperative complications, *n* (%)			
Any comorbidities	6 (5.7)	22 (7.0)	0.65
Prolonged air leak > 5 days	3 (2.9)	11 (3.5)	1.00
Pneumonia requiring antibiotics	1 (1.0)	5 (1.6)	1.00
Hemorrhage requiring intervention	1 (1.0)	2 (0.6)	1.00
Chylothorax	1 (1.0)	4 (1.3)	1.00
Arrhythmia	0 (0.0)	2 (0.6)	1.00
Vocal cord paralysis	0 (0.0)	1 (0.3)	1.00
Chest tube reinsertion	0 (0.0)	1 (0.3)	1.00
Readmission within 30 days	0 (0.0)	2 (0.6)	1.00
Number of dissected LNs, mean ± SD			
N1 LNs count	4.2 ± 3.1	3.5 ± 2.2	**0.02**
N2 LNs count	5.2 ± 2.7	4.8 ± 2.5	0.08
Total N1 + N2 LNs count	9.4 ± 4.4	8.3 ± 3.6	**0.03**
Number of dissected LN stations, mean ± SD			
N1 station count	2.5 ± 0.8	2.4 ± 0.9	0.07
N2 station count	3.2 ± 1.1	3.1 ± 0.9	0.45
Total N1 + N2 stations count	5.6 ± 1.4	5.4 ± 1.4	0.35

Continuous data are expressed as mean ± SD, and categorical data are shown as number (percentage). Bold indicates the statistically significant *p* value (*p* < 0.05). *RATS* robotic-assisted thoracoscopic surgery, *VATS* video-assisted thoracoscopic surgery, *SD* standard deviation, *LNs* lymph nodes.

In terms of the LN dissection, RATS harvested significantly increased number of N1 (4.2 ± 3.1 *vs* 3.5 ± 2.2, *p = *0.02) and total (9.4 ± 4.4 *vs* 8.3 ± 3.6, *p = *0.03) LNs compared with VATS. Meanwhile, the two groups had a comparable number of harvested N2 LNs (5.2 ± 2.7 *vs* 4.8 ± 2.5, *p = *0.08). Moreover, no difference was found in assessing N1 (2.5 ± 0.8 *vs* 2.4 ± 0.9, *p = *0.07), N2 (3.2 ± 1.1 *vs* 3.1 ± 0.9, *p = *0.45), and total (5.6 ± 1.4 *vs* 5.4 ± 1.4, *p = *0.35) LN stations between two surgical approaches.

### Long-term survival

**Fig. 3:**
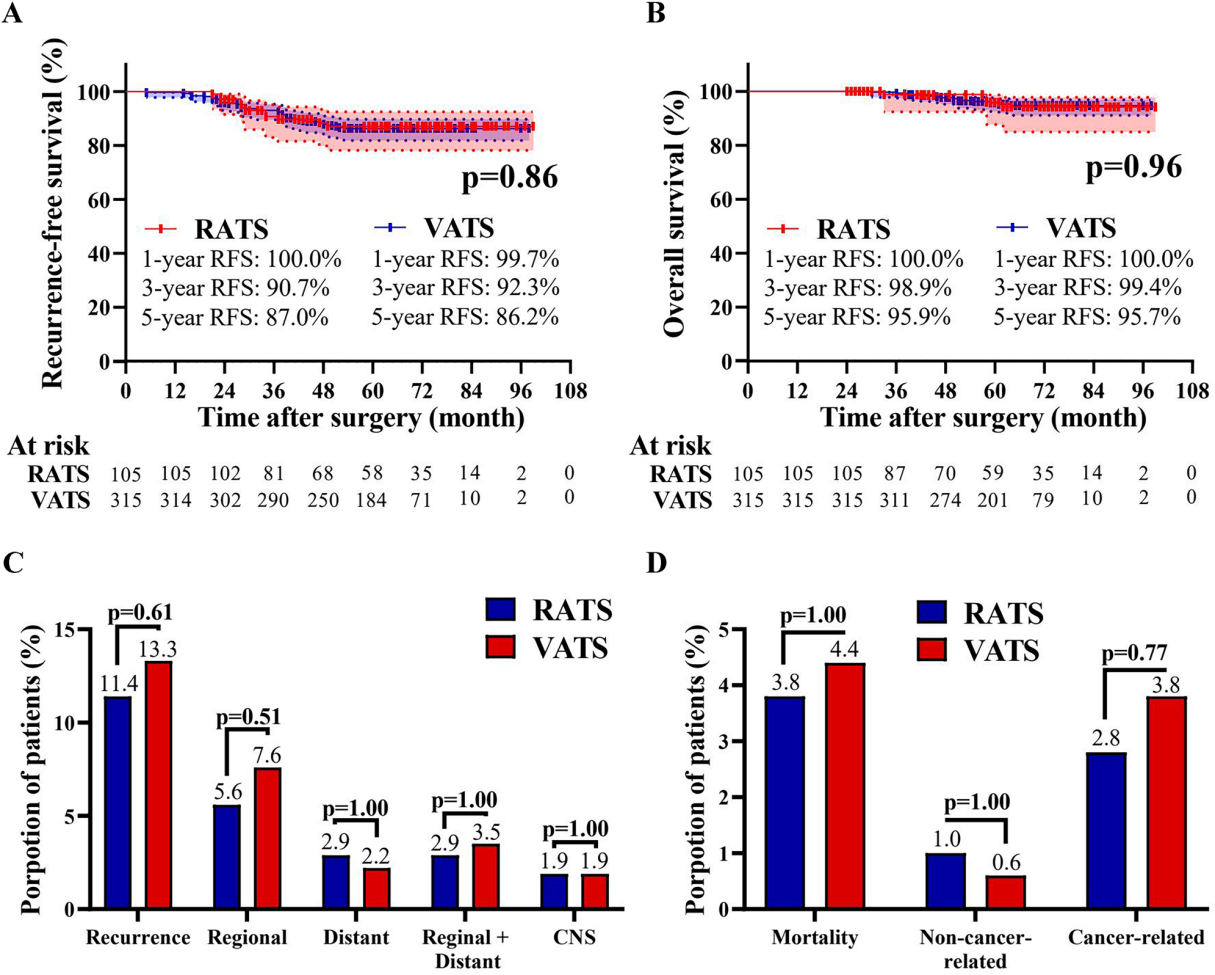
Kaplan–Meier analysis of long-term outcomes of NSCLC patients aged ≤ 35 years who underwent RATS or VATS. Comparison of RFS **A**, OS **B**, recurrence pattern **C**, and mortality pattern **D** between the RATS and VATS groups. Regional recurrence was defined as tumor recurrence or metastasis confined to the chest cavity, while distant recurrence was defined as tumor metastasis beyond the chest cavity. *RATS* robotic-assisted thoracoscopic surgery, *VATS* video-assisted thoracoscopic surgery, *RFS* disease-free survival, *OS* overall survival, *CNS* central nervous system

**Fig. 4:**
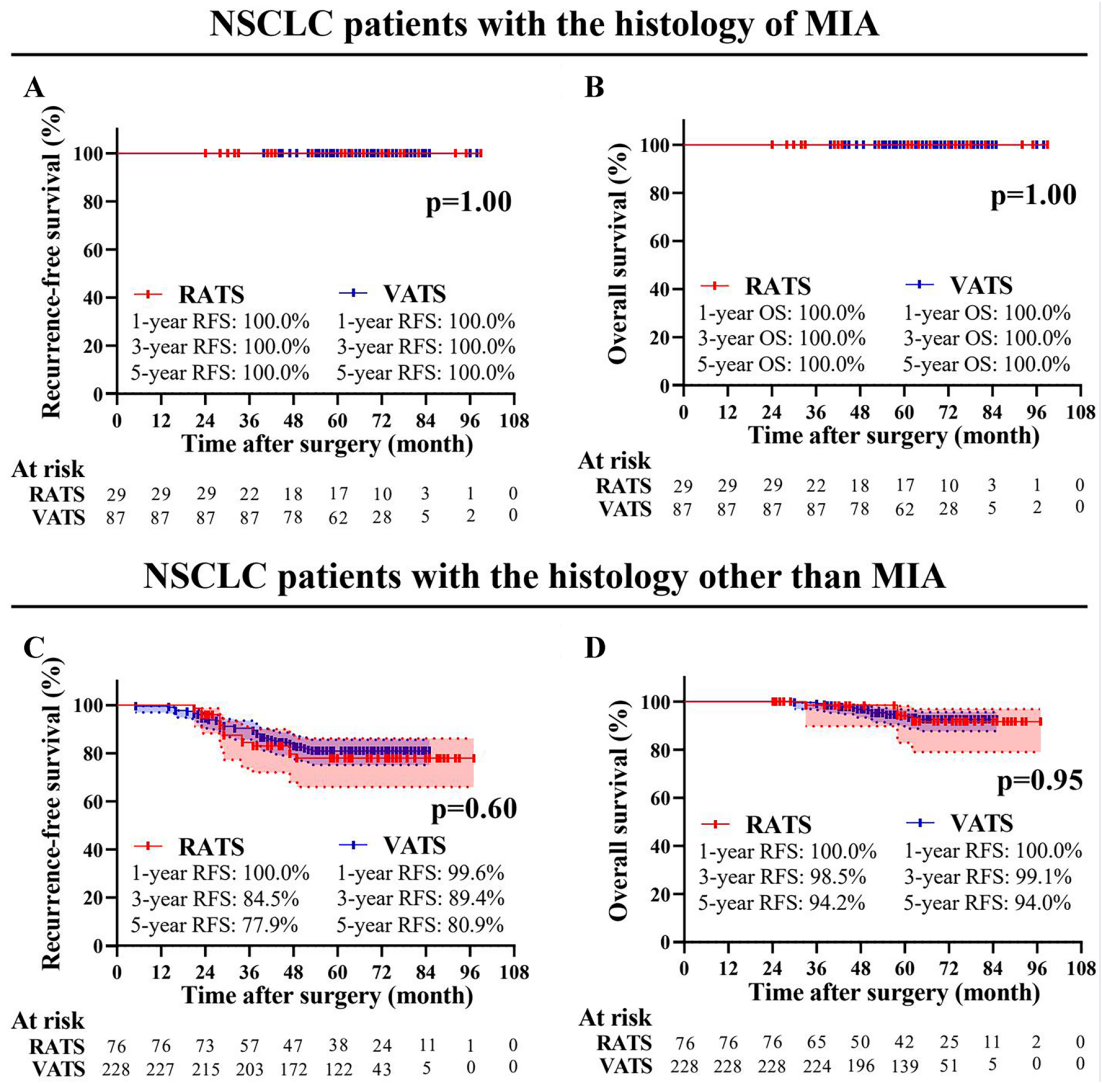
Subgroup analysis of NSCLC patients aged ≤ 35 years who underwent RATS or VATS. Comparison of RFS **A** and OS **B** between the RATS and VATS groups in patients with MIA. Comparison of RFS **C** and OS **D** between the RATS and VATS groups in NSCLC patients with histology other than MIA. *MIA* minimally invasive adenocarcinoma, *RATS* robotic-assisted thoracoscopic surgery, *VATS* video-assisted thoracoscopic surgery, *RFS* disease-free survival; *OS* overall survival

The median follow-up of RATS and VATS groups was 62 months [range 24–99 months] and 65 months [range 24–98 months], respectively. In the RATS group, the 5-year RFS and OS of patients were 87.0 and 95.9%, respectively. In the VATS group, the 5-year RFS and OS were 86.2 and 95.7%, respectively (Fig. 3A, B). The two surgical approaches achieved comparable RFS (*p = *0.86) and OS (*p = *0.96) profiles. Furthermore, patients who underwent RATS or VATS were associated with comparable recurrence and mortality patterns (Fig. 3C, D). Additionally, patients were divided into subgroups concerning the histology type (MIA and the others), age (18–30 and 31–35 years), and gender (male and female), and the analysis within the subgroups also suggested that RATS and VATS led to similar long-term outcomes (Fig. 4A–D, Supplementary Fig. 1A-D, Supplementary Fig. 2A-D). Finally, multivariable Cox hazard regression analysis revealed that the surgical method (RATS *vs* VATS) was not independently correlated with RFS [hazard ratio (HR) = 0.96, *p = *0.90, Supplementary Table 1) or OS (HR = 0.86, *p = *0.80, Supplementary Table 2). Meanwhile, gender, age, and history of smoke were also not independent predictors of prognosis (all *p > *0.05). Nevertheless, the advanced disease and LN metastasis were independently associated with the shortened DFS and OS.

## Discussion

The feasibility and efficacy of VATS have been well established, making VATS to be the most prevalent MIS technic in treating NSCLC. Nowadays, RATS is also increasingly applied for NSCLC and has been accepted to be safe and oncologically effective (Jin et al. 2022; Huang et al. 2021). However, the epidemiological characteristics of NSCLC determine that the vast majority of cases occur in elderly individuals aged over 60 years. Consequently, the perioperative outcomes and long-term survival data of RATS for young NSCLC cases remain unrevealed. In this study, we retrospectively compared the short- and long-term outcomes of RATS and VATS lobectomy in NSCLC patients aged 35 years or younger based on real-world practice, suggesting that RATS accelerated postoperative recoveries, assessed increased LNs, and achieved comparable intraoperative outcomes and long-term survival when compared with VATS.

Our results showed that RATS led to a shorter postoperative hospital stay than VATS, which potentially be attributed to the flexibility of the robot arms and high-quality surgical view, which enables a more precise resection and minimizes unnecessary damage and, thus, expedites postoperative recoveries of patients. Given current clinical practice trends, increasing publications compare perioperative recoveries between RATS and VATS, but conflicting results have been reported. Recently, three studies analyzing the Premiere database observed that RATS was associated with shorter postoperative hospitalization (Reddy et al. 2018; Oh et al. 2017; Kent et al. 2023). This superiority of RATS, however, was not found in the other two publications based on public databases (Louie et al. 2016; Veluswamy et al. 2020). Furthermore, our results showed that RATS and VATS led to similar duration and volume of chest tube drainage, concordant with most previous studies. Nevertheless, Jin et al*.* found an increased chest tube volume with RATS, possibly due to the advantages of RATS in accessing LNs which might damage the bronchial and lymphatic vessels connected with LNs (Jin et al. 2022). Finally, no surgical-related mortality and low incidence of blood transfusion were found in the RATS and VATS groups, suggesting that both surgical approaches are safe for lobectomy in treating very young NSCLC patients.

LN retrieval is a vital part of MIS lobectomy in treating NSCLC and an essential measurement of surgical quality. Numerous previous studies have determined the capacity of the robotic-assisted surgical system in LN assessment but have produced conflicting conclusions. Three studies suggested that RATS harvested higher numbers of LNs and LN stations than VATS (Jin et al. 2022; Nelson et al. 2019; Ma et al. 2021). Nevertheless, Kneuertz et al. and Guo et al*.* independently found that the two procedures exhibited comparable capacity in LN exanimation (Kneuertz et al. 2019; Guo, Ma et al. 2019). Our results indicated that RATS assessed more LNs, especially N1 LNs while examining similar N2 LNs and LN stations compared to VATS. In our real-world practice, the assessment may not further be performed for LNs that are likely to be uninvolved if difficulty in dissection is estimated and sufficient LNs and LN stations have been retrieved, especially for patients with clinically early-stage disease. Given this, a more convenient process is likely to improve the willingness of thoracic surgeons to harvest more LNs. As one of the most sophisticated, complex, and expensive operational equipment in the world, da Vinci Surgical System provides many natural superiorities, including 3D, high-definitional, and tenfold magnified surgical view, and robotic arms that can rotate freely in the chest cavity and exhibit excellent maneuverability and improved dexterity over traditional 2D VATS technic (Jin et al. 2022; Pan Tian et al. 2022; Kneuertz et al. 2019). This benefit offers operators great convenience in harvesting LNs around vessels and bronchi and helps surgeons to dissect LNs that VATS does not easily assess due to limited flexibility, which may explain the increased LNs assessed by RATS. Additionally, the varied operation experiences and the learning curve in performing RATS and VATS among different thoracic surgeons may also impact LN retrieval, which was hard to be controlled in a retrospective study. Nevertheless, VATS is still the most prevalent approach in our center, and therefore most surgeons are more experienced in performing VATS than RATS. Given this, the superiority in LN dissection of RATS over VATS may largely be attributed to the advantages of the robot-assisted surgical system. The increased LN dissection could prolong the surgical duration and damage the normal mediastinal lymphatic, vascular, and neurogenic tissues, and may, thus, increase intraoperative bleeding and result in comorbidities, including recurrent laryngeal nerve injury and chylothorax (Allen et al. 2006; Zhang et al. 2023). In our study, RATS did not increase postoperative complications when harvesting more LNs compared with VATS. Given this, RATS might be especially suitable for patients with a high LN metastasis risk demanding a more complete mediastinal and pulmonary LN assessment.

In terms of long-term outcomes, our results suggested that RATS and VATS achieved comparable 5 year RFS and OS in the young. These findings are consistent with and could complement several previous studies which enrolled older cases, suggesting that RATS might be oncologically effective for all-age resectable NSCLC patients (Montagne et al. 2022; Pan et al. 2022a, b; Merritt et al. 2022). The very young NSCLC patients usually have distinctive clinic-demographic and genomic features and are associated with a higher risk of suffering from multiple pulmonary lesions than older individuals (Viñal et al. 2021). Hence, identifying the optimal extent of resection to preserve more normal lung tissue for the potential multiple lung surgeries is of critical importance for this particular group of patients. Recently, two multi-center, noninferiority, phase 3 trials, namely JCOG0802/WJOG4607L and CALGB 140503 trials, showed that sub-lobectomy was not inferior to lobectomy, and thus might be considered one of the standard treatments for patients with a peripheral stage IA NSCLC with a tumor size ≤ 2 cm (Altorki et al. 2023; Saji et al. 2022). Moreover, two independent studies also found that patients with AIS/MIA were associated with 100% RFS during the follow-up for at least 5 years, regardless of the surgical method (lobectomy, segmentectomy, or wedge resection) (Yotsukura et al. 2021; Zhang et al. 2022). These crucial discoveries are expected to promote the prevalence of sub-lobectomy in treating early-stage NSCLC. In our study, most young patients had early-stage disease and met the criteria for sub-lobar resection proposed by the trials mentioned above. Therefore, further comparison of surgical-related outcomes and oncological efficacies of RATS versus VATS for sub-lobectomy in young NSCLC cases is essential to expand the scope of the application of RATS.

In the present study, most young NSCLC patients were associated with early-stage disease and achieved excellent 5-year survival outcomes. However, many previous publications have found distinct clinic-epidemiological features, indicating that NSCLC in the young may represent a more aggressive tumor, and most young cases had the late-stage (III–IV) disease at the first diagnosis and were associated with a poor prognosis (Duan et al. 2013; Subramanian et al. 2010; Galvez-Nino et al. 2020). Nevertheless, most included cases in these studies were diagnosed before 2016, and early-stage NSCLC is increasingly prevalent nowadays with the increased implementation of thin-section thoracic CT and development in diagnostic modalities. Meanwhile, more attention has been paid to physical health, and routine medical examination is becoming increasingly popular among young adults in recent years. Our study mostly identified young NSCLC patients undergoing surgery after 2016 and, thus, included more early-stage cases. Moreover, the present study excluded patients with stage T4/N3 disease or intra-pulmonary or distant metastasis, and therefore the vast majority of patients with advanced disease were excluded.

To the best of our knowledge, the present study is the first retrospective analysis comparing perioperative outcomes and long-term survival of RATS versus VATS lobectomy specified for very young NSCLC patients aged ≤ 35 years based on real-world practice. However, we have also acknowledged some limitations of the present study. The retrospective analysis usually leads to undiscovered patient selection bias and unbalanced case distribution. The massive difference in the size of included patients resulted in excluding many patients in the VATS group, and the potential selection bias may still exist despite PSM having been applied in the present study to balance the key confounding factors of patients. Moreover, the single-center analysis property of the present study limited the size of the case sample and weakened its representative, though the patient data were identified from one of the most famous and highest-volume medical centers in China. Therefore, further multi-center prospective research is necessary to validate the findings of our analysis.

## Conclusions

In conclusion, RATS exhibited superiorities over VATS in shorter postoperative hospital stay and more assessed LNs than VATS, and the two surgical approaches achieved similar long-term outcomes in treating NSCLC patients aged 35 years or younger.

## Supplementary Information

Below is the link to the electronic supplementary material.

Supplementary file1 (DOCX 541 KB)

Supplementary file2 (MP4 146915 KB)

## Data Availability

The data generated and analyzed during the present study are available from the corresponding authors, Prof. Huang J and Luo QQ, upon reasonable request.
