# Supplementary material for: Short- and long-term outcomes of robotic-assisted versus video-assisted thoracoscopic lobectomy in non-small cell lung cancer patients aged 35 years or younger: a real-world study with propensity score-matched analysis
Source: J Cancer Res Clin Oncol. 2023 May 30;149(12):9947–58. doi: 10.1007/s00432-023-04933-6 (PMC10423161; doi:10.1007/s00432-023-04933-6)
Supplement: Supplementary file 1 — Supplementary file1 (DOCX 541 KB) [file 432_2023_4933_MOESM1_ESM.docx]

**Supplementary Information**

**Title: Short- and long-term outcomes of robotic-assisted versus video-assisted thoracoscopic** **lobectomy in non-small cell lung cancer patients aged 35 years or younger: a real-world study with propensity-score matched analysis**

**Journal name: Journal of Cancer Research and Clinical Oncology**

**Hanbo Pan ^1#^, Jiaqi Zhang ^1#^, Yu Tian ^1#^, Ningyuan Zou ^1^, Hongda Zhu ^1^, Zenan Gu ^1^, Weiqiu Jin ^1^, Junwei Ning ^1, 2^, Long Jiang ^1^, Jia Huang ^1*^, Qingquan Luo ^1*^**

^1^ Shanghai Lung Cancer Center, Department of Thoracic Surgical Oncology, Shanghai Chest Hospital, Shanghai Jiao Tong University School of Medicine, Shanghai, China

^2^ Department of Thoracic Surgery, Tongren Hospital, Shanghai Jiao Tong University School of Medicine, Shanghai, China

Correspondence: Qingquan Luo

Email: luoqingquan@hotmail.com.

Co-correspondence: Jia Huang

Email: huangjiadragon@126.com.

ORCID: 0009-0003-0150-9511

*
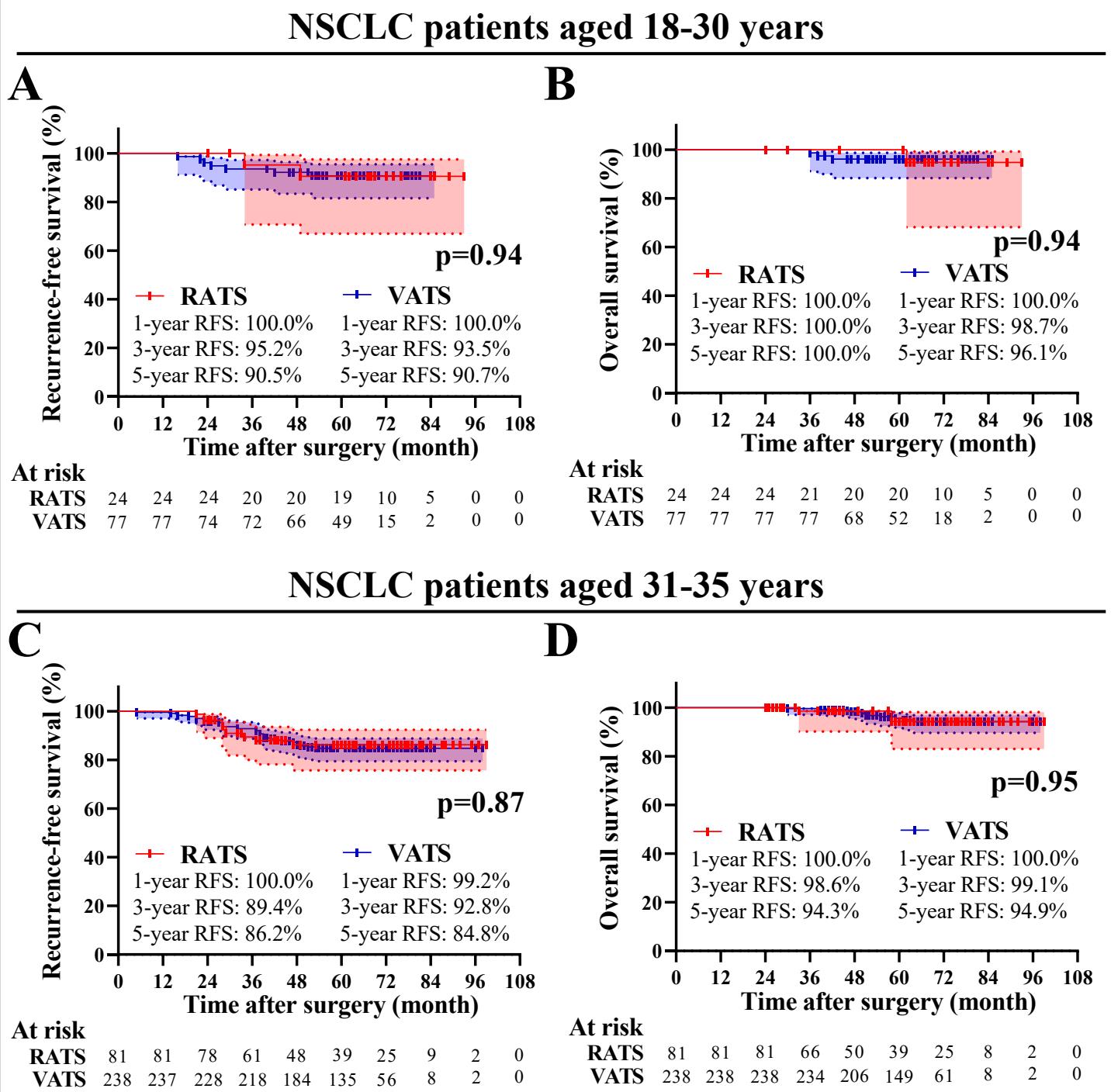
*

**Fig. S1** Subgroup analysis of long-term survival of NSCLC patients aged ≤35 years who underwent RATS or VATS concerning age. Comparison of RFS (A) and OS (B) between the RATS and VATS groups in patients aged 18-30 years. Comparison of RFS (C) and OS (D) between the RATS and VATS groups in patients aged 31-35 years. *NSCLC, non-small cell lung cancer; RATS, robot-assisted thoracoscopic surgery; VATS, video-assisted thoracoscopic surgery; RFS, recurrence-free survival; OS, overall survival.*

**
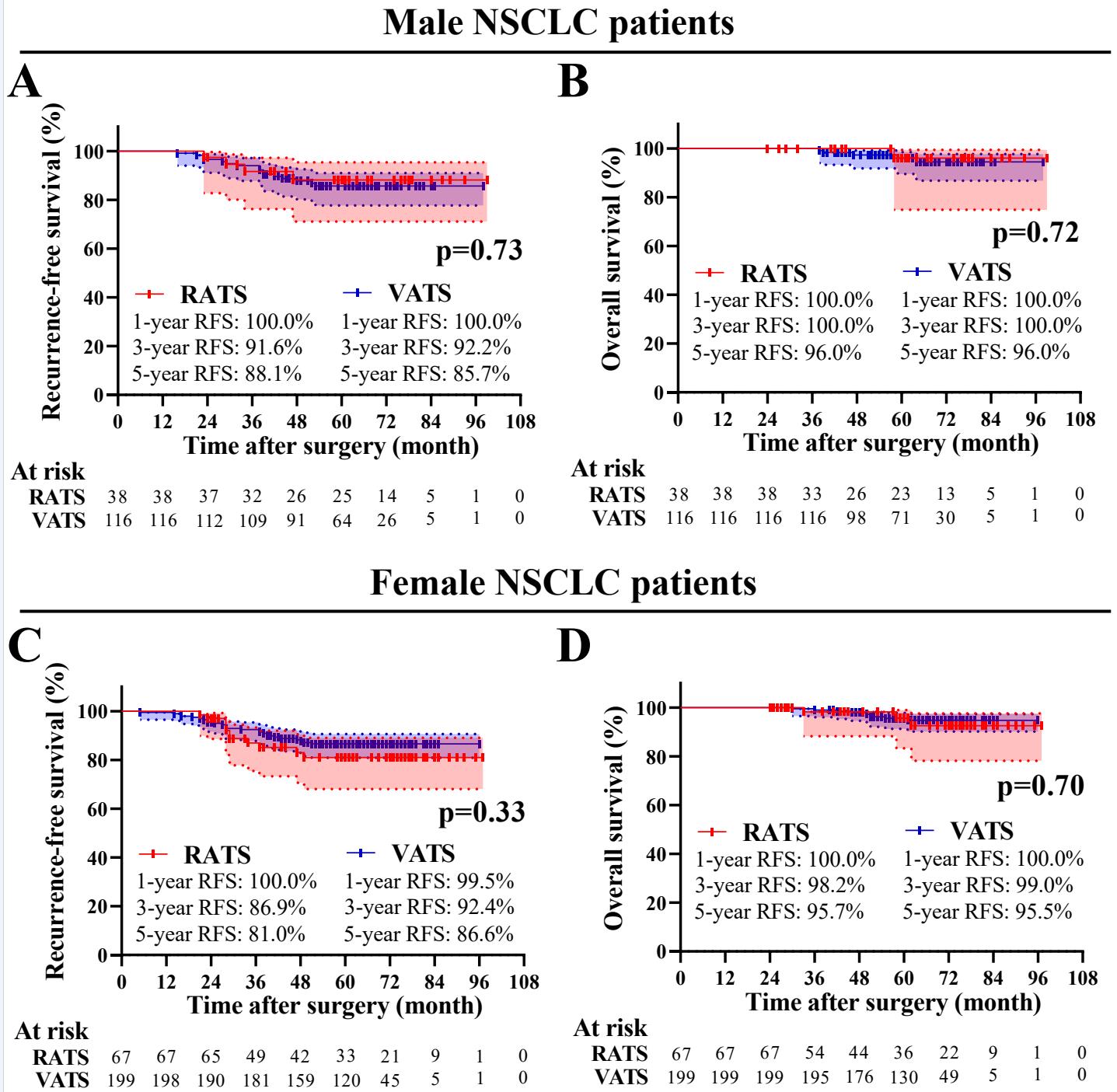
**

**Fig. S2** Subgroup analysis of long-term survival of NSCLC patients aged ≤35 years who underwent RATS or VATS concerning gender. Comparison of RFS (A) and OS (B) between the RATS and VATS groups in male patients. Comparison of RFS (C) and OS (D) between the RATS and VATS groups in female patients. *NSCLC, non-small cell lung cancer; RATS, robot-assisted thoracoscopic surgery; VATS, video-assisted thoracoscopic surgery; RFS, recurrence-free survival; OS, overall survival.*

**Table** **S1** Cox hazards regression model analysis for recurrence-free survival of patients receiving RATS or VATS

| Variables | Univariable | |  | Multivariable | |
| --- | --- | --- | --- | --- | --- |
|  | p-value | HR (95% CI) |  | p-value | HR (95% CI) |
| Surgical method (RATS *vs* VATS) | 0.55 | 0.84 (0.46-1.51) |  | 0.90 | 0.96 (0.51-1.80) |
| Gender (male *vs* female) | 0.16 | 0.69 (0.41-1.16) |  | 0.81 | 1.07 (0.60-1.91) |
| Age (≤30 *vs* >30 years) | 0.51 | 1.24 (0.66-2.34) |  | 0.24 | 1.47 (0.77-2.79) |
| History of smoke (never *vs* ever) | 0.90 | 1.07 (0.39-2.95) |  | 0.59 | 0.75 (0.26-2.15) |
| Pathological stage (II-III *vs* I) | **<0.01** | 12.79 (7.52-21.73) |  | **<0.01** | 4.83 (2.22-10.54) |
| LN status (positive *vs* negative) | **<0.01** | 16.76 (9.71-28.92) |  | **<0.01** | 5.95 (2.63-13.45) |

Bold indicates the statistically significant p-value (p<0.10). *HR, hazard Ratio; CI, Confidence Interval; RATS, robot-assisted thoracoscopic surgery; VATS, video-assisted thoracoscopic surgery; MIA, minimally invasive adenocarcinoma; LN, lymph node.*

**Table S2** Cox hazards regression model analysis for overall survival of patients receiving RATS or VATS

| Variables | Univariable | |  | Multivariable | |
| --- | --- | --- | --- | --- | --- |
|  | p-value | HR (95% CI) |  | p-value | HR (95% CI) |
| Surgical method (RATS *vs* VATS) | 0.92 | 0.95 (0.31-2.87) |  | 0.80 | 0.86 (0.26-2.81) |
| Gender (male *vs* female) | 0.21 | 0.56 (0.22-1.40) |  | 0.80 | 0.87 (0.32-2.41) |
| Age (≤30 *vs* >30 years) | 0.75 | 1.20 (0.39-3.64) |  | 0.60 | 1.35 (0.43-4.20) |
| History of smoke (never *vs* ever) | 0.78 | 1.34 (0.18-10.05) |  | 0.58 | 0.55 (0.06-4.70) |
| Pathological stage (II-III *vs* I) | **<0.01** | 24.48 (9.40-63.74) |  | **0.04** | 4.94 (1.11-21.90) |
| LN status (positive *vs* negative) | **<0.01** | 31.79 (12.24-82.55) |  | **<0.01** | 10.34 (2.23-48.09) |

Bold indicates the statistically significant p-value (p<0.10). *HR, Hazard Ratio; CI, Confidence Interval; RATS, robot-assisted thoracoscopic surgery; VATS, video-assisted thoracoscopic surgery; MIA, minimally invasive adenocarcinoma; LN, lymph node.*

**Video S1** The representative operation video of RATS lobectomy. A 35-year-old man with ADC underwent radical left lower lobectomy with systemic mediastinal LN assessment by using the da Vinci Surgical System. The inferior pulmonary ligament and 9^th^ LNs were dissected, followed by the dissection of hilar structures and LNs, Then, the 7^th^ LNs were harvested, and the inferior pulmonary vein was dissected. After this, the left lower lobe bronchus and inferior lobar artery were dissected. Finally, upper mediastinal LNs were assessed. *RATS, robot-assisted thoracoscopic surgery; ADC, adenocarcinoma; LN, lymph node.*
